# Non Digestible Oligosaccharides Modulate the Gut Microbiota to Control the Development of Leukemia and Associated Cachexia in Mice

**DOI:** 10.1371/journal.pone.0131009

**Published:** 2015-06-22

**Authors:** Laure B. Bindels, Audrey M. Neyrinck, Nuria Salazar, Bernard Taminiau, Céline Druart, Giulio G. Muccioli, Emmanuelle François, Christophe Blecker, Aurore Richel, Georges Daube, Jacques Mahillon, Clara G. de los Reyes-Gavilán, Patrice D. Cani, Nathalie M. Delzenne

**Affiliations:** 1 Metabolism and Nutrition Research Group, Louvain Drug Research Institute, Université catholique de Louvain, Brussels, Belgium; 2 Fundamental and Applied Research for Animal and Health (FARAH), Faculty of Veterinary Medicine, University of Liège, Sart Tilman, Liège, Belgium; 3 Bioanalysis and Pharmacology of Bioactive Lipids lab, Louvain Drug Research Institute, Université catholique de Louvain, Brussels, Belgium; 4 Department of Chemistry and Bio-industry, Gembloux Agro-Bio Tech, University of Liège, Gembloux, Belgium; 5 Laboratory of Food and Environmental Microbiology, Earth and Life Institute, Université catholique de Louvain, Louvain-la-Neuve, Belgium; 6 Department of Microbiology and Biochemistry of Dairy Products, Instituto de Productos Lácteos de Asturias, (IPLA-CSIC), Villaviciosa, Asturias, Spain; 7 Walloon Excellence in Life sciences and BIOtechnology (WELBIO), Louvain Drug Research Institute, UCL, B-1200 Brussels, Belgium; Kobe University, JAPAN

## Abstract

We tested the hypothesis that changing the gut microbiota using pectic oligosaccharides (POS) or inulin (INU) differently modulates the progression of leukemia and related metabolic disorders. Mice were transplanted with Bcr-Abl-transfected proB lymphocytes mimicking leukemia and received either POS or INU in their diet (5%) for 2 weeks. Combination of pyrosequencing, PCR-DGGE and qPCR analyses of the 16S rRNA gene revealed that POS decreased microbial diversity and richness of caecal microbiota whereas it increased *Bifidobacterium* spp., *Roseburia* spp. and *Bacteroides* spp. (affecting specifically *B*. *dorei*) to a higher extent than INU. INU supplementation increased the portal SCFA propionate and butyrate, and decreased cancer cell invasion in the liver. POS treatment did not affect hepatic cancer cell invasion, but was more efficient than INU to decrease the metabolic alterations. Indeed, POS better than INU delayed anorexia linked to cancer progression. In addition, POS treatment increased acetate in the caecal content, changed the fatty acid profile inside adipose tissue and counteracted the induction of markers controlling β-oxidation, thereby hampering fat mass loss. Non digestible carbohydrates with prebiotic properties may constitute a new nutritional strategy to modulate gut microbiota with positive consequences on cancer progression and associated cachexia.

## Introduction

Over the past decade, gut microbiota has appeared as a fully-fledged component involved in host energy homeostasis. Recent studies have highlighted that changes in gut microbial ecosystem are associated with specific diseases and life steps, such as inflammatory bowel diseases, obesity, diabetes, autism, pregnancy and ageing [1, 2]. Thus developing tools able to influence the composition and metabolic activity of the gut microbiota—such as prebiotics—might lead to innovative therapeutic approaches.

We have recently proposed to define prebiotics as nondigestible compounds that, through its metabolization by microorganisms in the gut, modulates composition and/or activity of the gut microbiota, thus conferring a beneficial physiological effect on the host [3]. One of the most studied prebiotics is inulin-type fructans (ITF) [4–7]. New prebiotic compounds arouse a high therapeutic interest because different prebiotics could differentially impact the gut microbial ecosystem, thereby leading to specific health benefits in pathological contexts.

Prebiotic candidates include pectine derivatives. Pectins are soluble dietary fibres which exert physiological effects on the gastrointestinal tract such as delayed gastric emptying, reduced transit time and reduced glucose absorption [8, 9]. Pectins reach the large intestine and are fermented by the gut microbiota [10, 11]. Subfractions of pectins, called pectic oligosaccharides (POS), are interesting prebiotic candidates despite the fact that they are less bifidogenic than short-chain ITF. POS consist mainly of partially acetylated rhamnogalacturonan oligosaccharides and partially methyl-esterified/acetylated homogalacturonan oligosaccharides, the degree of methylation playing an important role in the fermentation properties [10]. An *in vitro* study revealed that six types of pectin-derived oligosaccharides increase the concentration of short chain fatty acids (SCFA) (especially acetate and propionate) and the *Bacteroides-Prevotella* group in the human fecal microbiota [12].

We have recently shown that the composition of the gut microbiota is altered in leukemic mice suffering from cachexia [13]. Cachexia is a complex metabolic disorder associated with an underlying disease and characterized by muscle atrophy and loss of fat mass. Cancer cachexia is associated with reduced quality of life and lifespan. Thirty nine percent of patients with acute non lymphocytic leukemia experience weight loss in the six month-period preceding chemotherapy [14]. Weight loss and anorexia have also been described in acute childhood lymphoblastic leukemia and in chronic myeloid leukemia [15, 16]. Cancer cachexia currently remains an unmet medical need, for which a multimodal package approach, including nutritional intervention, is recommended [17–19]. Interestingly, oral administration of a probiotic mixture to leukemic mice reduced systemic inflammation and muscle atrophy markers, two key components of the cachexia syndrome [13]. Furthermore, administration of ITF was accompanied by a reduced accumulation of leukemic cells in the liver [20].

In view of these studies, we hypothesized that POS prepared from beet pulp are original colonic nutrients able to influence the progression of cancer cachexia. The prebiotic potency of the POS extract was compared to the inulin’s one, in a validated mouse model of acute leukemia and cachexia. Gut microbiota-host crosstalk was characterized through the analysis of gut microbial members and metabolites, as well as the assessment of morphological, biochemical and molecular host parameters.

## Materials and Methods

### Cell culture

ProB lymphocytes (BaF3) cell line transfected with Bcr-Abl was a gift from Dr. K. Bhalla in 2007 (MCG Cancer Center, Medical College of Georgia, Augusta, GA, USA). The generation of these cells is described in detail elsewhere [21, 22]. The BaF3 cells were maintained in RPMI1640 medium supplemented with 10% fetal bovine serum (PAA clone, PAA, Pasching, Austria), streptomycin 100 μg/ml, penicillin 100 IU/ml, and 1% of non-essential amino acids solution (Gibco, Inchinnan, Scotland) at 37°C in humidified 5% CO_2_ atmosphere.

### Animals and diets

Male BALB/c mice (8-week-old, Charles River Laboratories, Belgium) were housed two mice per cage in a 12 h light/dark cycle. After an acclimatization period of 1 week, the mice were divided into 4 groups (n = 8–9, except for controls n = 6): a control group (CT) receiving a saline injection and fed with a synthetic diet (AIN93M, Research Diet, New Brunswick, NJ, USA), a group receiving BaF3 cells and fed with AIN93M diet (BaF), a group receiving BaF3 cells and fed with AIN93M diet supplemented with INU (5% INU (w/w); BaF-INU), and a group receiving BaF3 cells and fed with AIN93M diet supplemented with POS (5% POS (w/w); BaF-POS). Cosucra Groupe Warcoing (Belgium) supplied INU extracted from chicory roots (Fibruline) with 90% degree of purity (10% free glucose, fructose and sucrose) and an average degree of polymerization of 10. The degree of polymerization and sugar compositions of POS are detailed in S1 Table.

On day 0, BaF3 cells (10^6^ cells) or vehicle were injected in the tail vein of the anesthetized mice. INU or POS were administered since day 1 post-injection. The food intake was monitored taking into account spillage. The caloric intake was calculated assuming the following caloric value, i.e. 3.85 kcal/g for CT and BaF, and 3.76 kcal/g for BaF-INU and BaF-POS. Total fat mass was determined using a 7.5MHz Time domain-Nuclear magnetic resonance (LF50 minispec, Bruker, Germany). After 15 days, mice were anaesthetized with isoflurane gas (Abbot, Ottignies, Belgium). Portal blood was collected, centrifuged (13000 g, 3 min) and the serum was stored at -80°C. Mice were sacrificed by cervical dislocation. The caecal content and tissue, the spleen, the liver, adipose tissues and muscles were collected, weighed, frozen in liquid N_2_ and stored at -80°C.

### Ethics statement

The agreement of the animal experiments performed in this study was given by the local Ethical Committee under the specific number 2010/UCL/MD/022. Housing conditions were as specified by the Belgian Law of 29 May 2013, on the protection of laboratory animals (Agreement LA 1230314). All surgeries were performed under anesthesia (isoflurane gas or ketamine/xylazine) and all efforts were made to minimize suffering. Condition of the animals was monitored every day in the late stage of the disease (from day 11 to day 15), this timeframe is based on previous observations [13].

### Gut microbiota analyses

Genomic DNA was extracted from the caecal content using a QIAamp DNA Stool Mini Kit (Qiagen, Hilden, Germany) according to the manufacturer’s instructions, including a bead-beating step. 454-pyrosequencing, Denaturing Gradient Gel Electrophoresis (DGGE) and qPCR were performed as descripted in S1 File.

### Short chain fatty acids analysis

Portal SCFA were quantified using high performance liquid chromatography coupled to mass spectrometry (HPLC-MS) following extraction from serum and subsequent derivatisation as previously described [20].

Cell-free supernatants from the homogenized caecal samples were filtered through 0.2 μm filters and were used for quantification of SCFA by GC. A chromatographic system composed of two 6890N GC (Agilent Technologies Inc., Palo Alto, CA) connected to a FID and a MS 5973N detector (Agilent) was used for quantification and identification of SCFA as described previously [23].

### Tissue mRNA analyses

Total RNA was isolated from tissues using the TriPure isolation reagent kit (Roche Diagnostics, Penzberg, Germany). For adipose tissue, RNA quality was checked using a Agilent 2100 Bioanalyzer (Agilent Technologies, Santa Clara, CA) with a quality threshold at 6. Complementary DNA was prepared by reverse transcription of 1 μg total RNA using the Kit Reverse Transcription System (Promega, Madison, WI). Real-time polymerase chain reaction (PCR) was performed with a StepOnePlus Real-Time PCR System and software (Applied Biosystems, Den Ijssel, The Netherlands) using SYBR Green (Applied Biosystems and Eurogentec, Verviers, Belgium) for detection. All samples were run in duplicate in a single 96-well reaction plate, and data were analyzed according to the 2-ΔΔCT method. The purity of the amplified product was verified by analyzing the melting curve performed at the end of amplification. The ribosomal protein L19 (RPL19) gene was chosen as a reference gene. Primer sequences are presented in S2 Table.

### Blood parameters

Blood glucose concentration was determined using a glucose meter (Roche Diagnostic, Meylan, France. Serum triglycerides, glycerol and non-esterified fatty acid concentrations were measured using kits coupling enzymatic reaction and spectrophotometric detection of reaction end-products (Diasys Diagnostic and Systems, Holzheim, Germany; Randox Laboratories Ltd., United Kingdom; Sigma, Saint-Louis, USA, respectively).

### Lipid analysis in the liver and the adipose tissue

Triglycerides and cholesterol were measured in the liver tissue after extraction with chloroform–methanol as previously described [24]. Fatty acid profile was measured in subcutaneous adipose tissue using gas chromatrography coupled to ion flame detector as previously described [25].

### Statistical analyses

Results are presented as the mean ± SEM, or Whiskers plot with minimum and maximum, for bacteria. Statistical significance of differences between groups was assessed by one-way ANOVA followed by post hoc Tukey’s multiple comparison test. The daily caloric intake and fat mass evolution were analysed by two-way ANOVA for repeated measures, followed by Bonferroni post hoc test. P < 0.05 was considered statistically significant. Statistical analyses were performed using GraphPad Prism 5.0 (GraphPad Software, San Diego, CA) and JMP 8.0.1 (SAS Institute, Inc., Cary, NC).

## Results

### POS supplementation induces drastic changes in microbial diversity and populations

#### Analysis of gut bacterial populations by pyrosequencing

**Fig 1:**
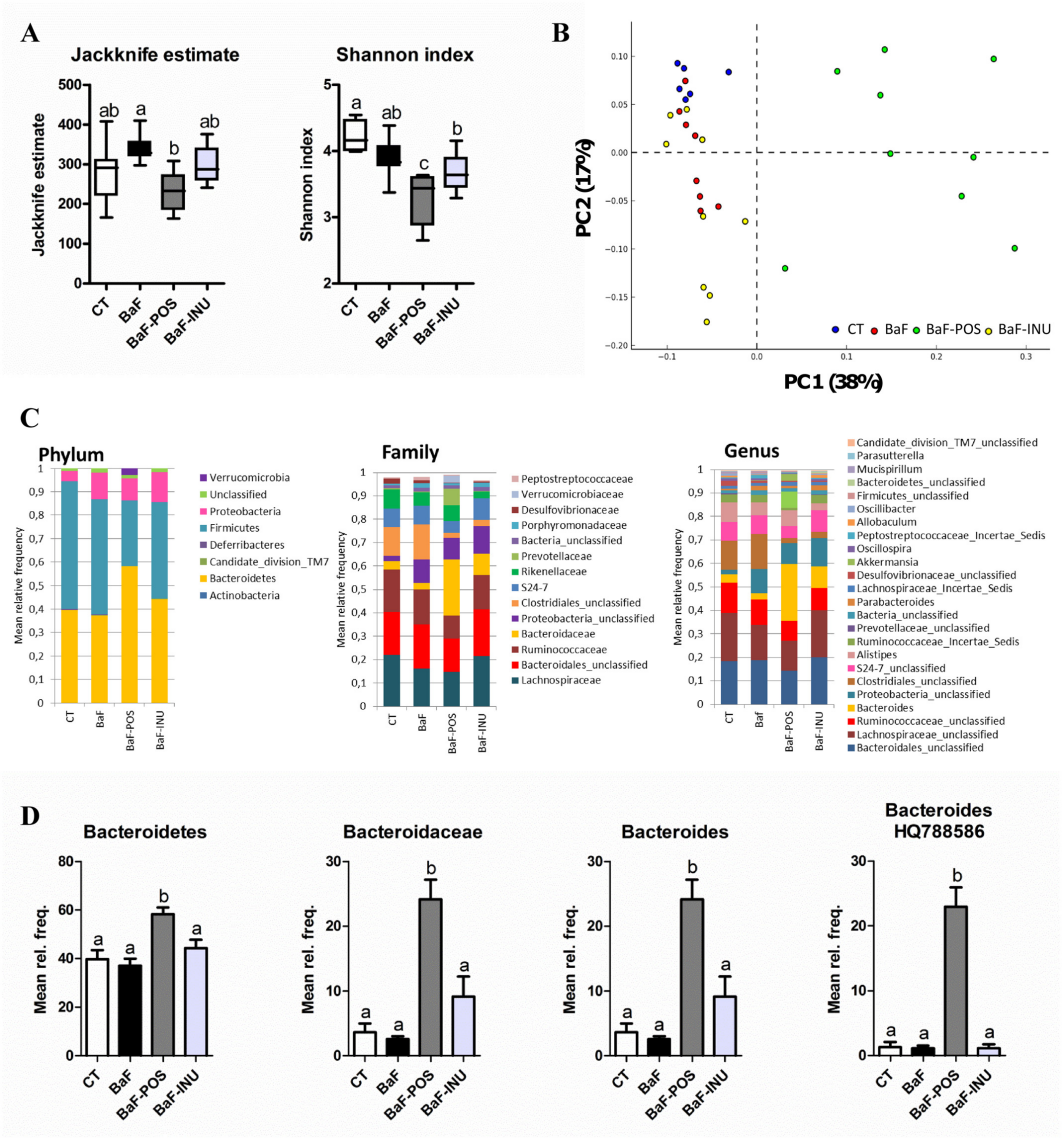
Changes in microbial diversity and populations in the caecal content, assessed by 454-pyrosequencing. Microbial diversity indexes (A). Principal component analysis based on the relative abundance distribution at the species level (B). Relative abundances of bacterial taxa accounting for more than 1%, at the phylum, family and genus levels (C). Relative abundances of the Bacteroidetes phylum, *Bacteroidaceae* family, *Bacteroides* genus and species-like *Bacteroides* HQ788586 (D). Data with different superscript letters are significantly different.

The BaF-POS mice exhibited a decreased caecal microbial diversity compared to the BaF mice (Fig 1A). The differences within the intestinal microbial ecosystem between the treatment groups were first assessed by principal component analysis (PCA) of the relative abundances at the genus level (Fig 1B) and 38% of the total variation was explained by the first PCA axis. Indeed, a distinct cluster was observed for mice receiving POS supplementation. To assess specific changes in intestinal microbiota, we compared the relative abundance of bacterial taxa between treatment groups (Fig 1C and S3 Table). We observed a significant phylum-wide shift from Firmicutes to Bacteroidetes upon POS supplementation. At the family level, the abundance of *Desulfovibrioceae* significantly decreased in BaF mice vs CT mice. This effect was even more pronounced under POS or INU supplementation. Several important changes were observed only after POS supplementation such as a decrease in *Ruminococaceae* and S24-7 families and in contrast a 31- and 9-fold increase in *Prevotellaceae* and *Bacteroidaceae*, respectively, two major families belonging to Bacteroidetes phylum. At a lower taxonomic level, the most prominent difference was obtained with POS supplementation inducing a huge increase in *Bacteroides* genus (reaching more than 20% of abundance). In contrast, INU supplementation led to a 2-fold decrease in *Alistipes* spp. belonging to the *Rikenellaceae* family. Finally, we determined that the most prominent increase at the OTU level after POS treatment corresponded to *Bacteroides* HQ788586 that gave 99% homology with *B*. *dorei*. This 20-fold increase of *B*. *dorei* impacts on the relative abundances of the genus, family and phylum to which this microbe belongs (Fig 1D).

#### Analysis of gut bacterial populations by DGGE

The DGGE fingerprints for total bacteria indicated 4 separate clusters corresponding to the 4 treatments. Furthermore, PCR-DGGE of the 16S rRNA gene revealed qualitative changes in *Bacteroides* genus for the POS group (S1 Fig). The principal species found in the DGGE profiles were identified and summarized in S4 Table. The enrichment of band 2 and band 3, identified as *B*. *dorei/B*. *vulgatus* and *Prevotella* spp. respectively, were evidenced after POS treatment.

#### Analysis of gut bacterial populations by qPCR

**Fig 2:**
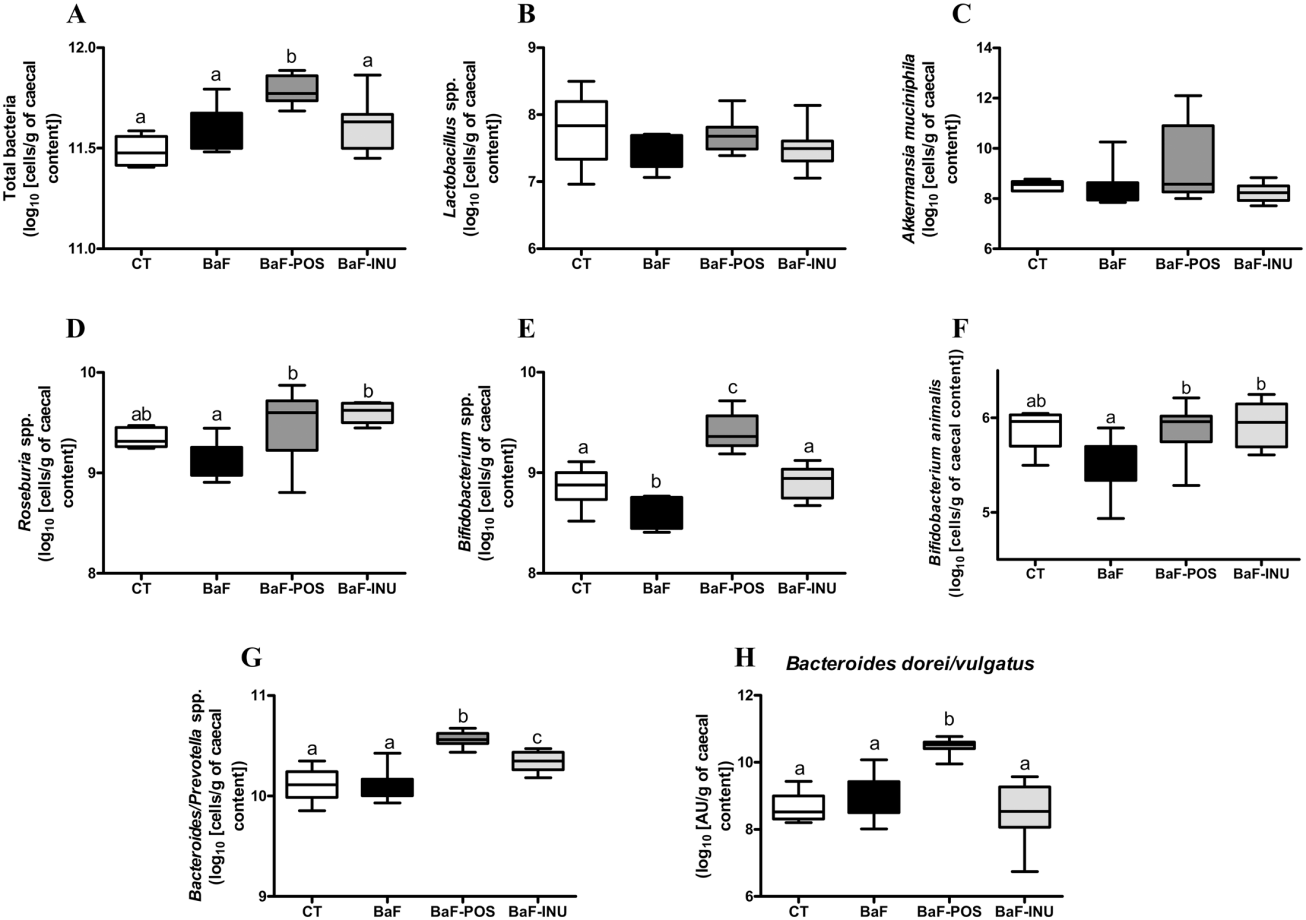
qPCR analysis of the caecal luminal microbiota. Levels of total bacteria (A), *Lactobacillus* spp. (B), *Akkermansia muciniphila* (C), *Roseburia* spp. (D), *Bifidobacterium* spp. (E), *Bifidobacterium animalis* (F), *Bacteroides/Prevotella* spp. (G) and *Bacteroides dorei/ Bacteroides vulgatus* (H). Data with different superscript letters are significantly different.

A qPCR approach was used to confirm results obtained by pyrosequencing and PCR-DGGE, and to detect other species important to take into account in the prebiotic context and that were not (or weakly) detected through pyrosequencing such as *Bifidobacterium* spp. (in particular *Bifidobacterium animalis*), *Lactobacillus* spp., *Roseburia* spp. and *Akkermansia* spp. (Fig 2). Among those bacteria, *Bifidobacterium* spp. was the only genus observed to be significantly decreased by the BaF injection. In contrast to INU, POS administration increased total bacteria. Interestingly, the increase of *Bacteroides/Prevotella* spp. and *Bifidobacterium* spp. was greater with POS treatment than INU treatment. Both treatments increased caecal content of *Roseburia* spp. and *B*. *animalis* to the same extent. Of note, the abundance of *Akkermansia* spp., even if it largely increased in two mice in the POS group, was not significantly affected by the dietary treatments. Importantly, we confirmed the specific increase of *B*. *dorei/B*. *vulgatus* after POS administration. In this study, pyrosequencing targeted a different hypervariable region of the 16S rRNA gene than the PCR-DGGE and qPCR analysis, allowing us to differentiate *B*. *dorei* from *B*. *vulgatus* and draw the conclusion that POS administration increases *B*. *dorei*.

### POS and INU supplementation differently modulates the profile of short chain fatty acids

**Fig 3:**
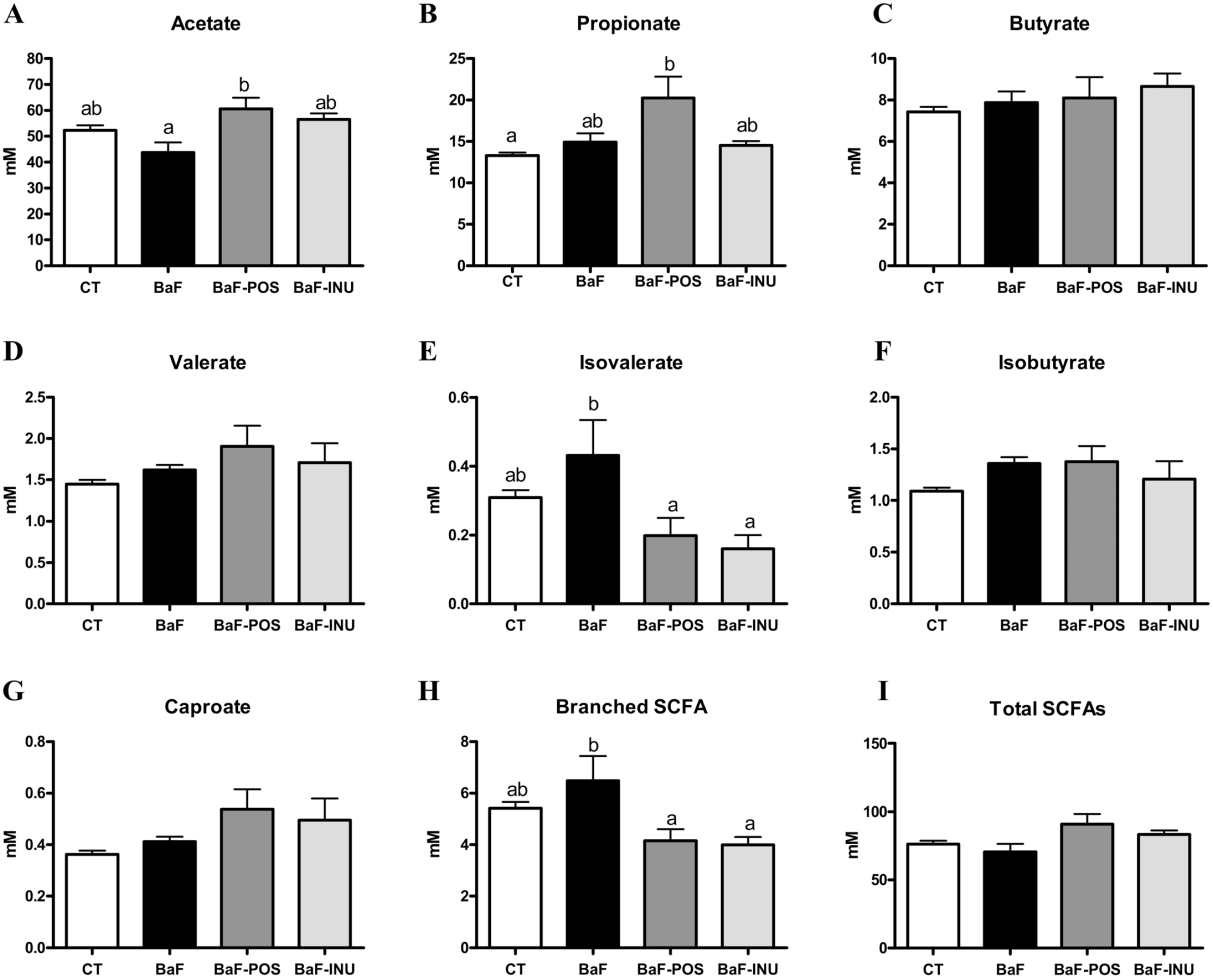
Short chain fatty acid (SCFA) profile in the caecal content. Acetate (A). Propionate (B). Butyrate (C). Valerate (D). Isovalerate (E). Isobutyrate (F). Caproate (G). Branched SCFA (H). Total SCFAs (I). Data with different superscript letters are significantly different.

Transplantation of BaF3 cells in mice did not modify levels of SCFA in the caecal content (Fig 3) or in the portal blood (Table 1). Supplementation with INU induced minor changes of SCFA in the caecum, namely a reduction in isovalerate (Fig 3E). However, it almost doubled propionate and butyrate levels in the portal blood (Table 1). POS increased acetate and lowered isovalerate in the caecal content, but did not significantly modify portal SCFA (Fig 3A and 3E, and Table 1). Both POS and INU reduced caecal branched SCFA, which are associated with negative end products of protein fermentation [4].

**Table 1:** Body and tissue weight, portal serum parameters and hepatic lipids.

	CT	BaF	BaF-POS	BaF-INU
**Body and organ weights**				
Body weight gain (g)	1.27 ± 0.18^a^	2.81 ± 0.37^b^	2.77 ± 0.29^b^	2.11 ± 0.16^ab^
Liver (g)	1.37 ± 0.09^a^	3.04 ± 0.21^b^	2.83 ± 0.16^b^	3.10 ± 0.22^b^
Spleen (g)	0.08 ± 0.01^a^	1.05 ± 0.05^b^	1.01 ± 0.03^b^	1.08 ± 0.05^b^
Subcutaneous adipose tissue (g)	0.44 ± 0.06^a^	0.25 ± 0.03^bc^	0.33 ± 0.02^ac^	0.21 ± 0.02^b^
Visceral adipose tissue (g)	0.22 ± 0.03^a^	0.14 ± 0.02^b^	0.17 ± 0.01^ab^	0.12 ± 0.01^b^
Gastrocnemius muscle (mg)	132.0 ± 4.4	131.7 ± 3.0	130.3 ± 2.4	130.2 ± 2.6
Soleus muscle (mg)	5.9 ± 0.2	6.1 ± 0.3	6.2 ± 0.2	5.8 ± 0.2
Caecal tissue (mg)	79.0 ± 4.5^ab^	70.4 ± 3.3^a^	88.8 ± 3.0^b^	93.3 ± 5.2^b^
Caecal content (mg)	163.9 ± 12.4^a^	89.8 ± 11.5^b^	114.1 ± 13.1^ab^	132.1 ± 17.2^ab^
**Serum parameters**				
Glycerol (mM)	0.17 ± 0.02	0.19 ± 0.02	0.17 ± 0.01	0.18 ± 0.02
Non esterified fatty acids (mM)	0.21 ± 0.05	0.28 ± 0.03	0.34 ± 0.03	0.31 ± 0.03
Triglycerides (mM)	1.01 ± 0.13	1.31 ± 0.07	1.27 ± 0.12	0.98 ± 0.10
Glycemia (mg/dl)	135 ± 8^a^	100 ± 14^ab^	96 ± 9^ab^	87 ± 7^b^
Acetate (μM)	188 ± 40	311 ± 100	423 ± 66	481 ± 126
Propionate (μM)	15.5 ± 2.8^ab^	6.8 ± 1.5^a^	15.5 ± 2.3^ab^	24.7 ± 3.9^b^
Butyrate (μM)	34.7 ± 5.9^ab^	27.1 ± 4.7^a^	30.7 ± 3.6^a^	63.1 ± 11.6^b^
**Hepatic lipids**				
Triglycerides (nmol/mg prot.)	288 ± 39	309 ± 36	320 ± 29	316 ± 38
Cholesterol (nmol/mg prot.)	99 ± 8	114 ± 8	110 ± 9	113 ± 19

Data are mean ± SEM. Data with different superscript letters are significantly different.

### POS supplementation improves metabolic phenotype to a higher extent than INU

**Fig 4:**
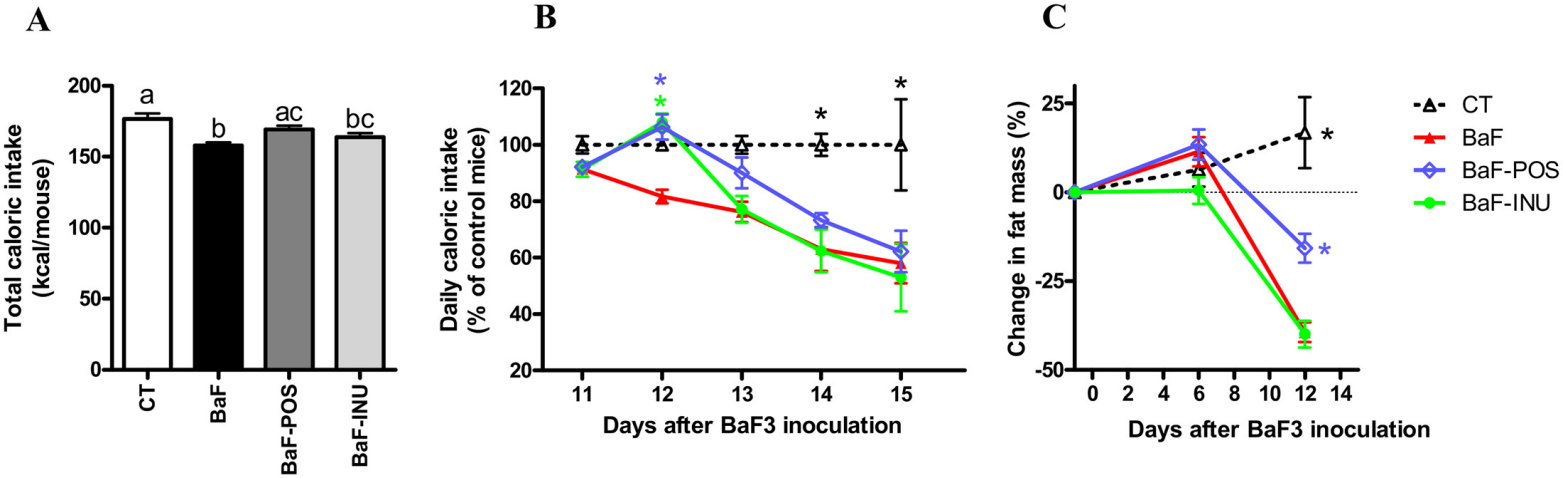
Caloric intake and fat mass evolution. Total caloric intake from the day of BaF inoculation to the necropsy (day 15) (A). Daily caloric intake per mice from day 11 to day 15 (B). Evolution of fat mass (C). Data with different superscript letters are significantly different.

The total caloric intake was lower in BaF mice than CT mice (Fig 4A). The BaF group exhibited a reduced food intake from day 11 to day 15 (Fig 4B). Anorexia induced by the transplantation of BaF cells was accompanied by a loss of total fat mass linked to cancer progression (Fig 4C). Supplementation with POS significantly delayed the fall of food intake to a higher extent than INU (Fig 4A and 4B). Moreover, POS treatment reduced fat mass loss by about 50% (Fig 4 *C*), whereas INU supplementation had no effect on fat mass. BaF transplantation decreased adipose tissue weight (subcutaneous and visceral) without affecting the muscle weight (soleus and gastrocnemius) (Table 1) in accordance with the well-established kinetics of this model [13]. POS treatment partly maintained adiposity, confirming the total fat mass analysis performed on live animals.

Transplantation of BaF cells resulted in an increase of liver and spleen weights, likely due to the accumulation of BaF cells in these organs (Table 1). Dietary treatments did not change liver and spleen weights. The weight of caecal content and tissue was decreased after BaF injection (Table 1). Both dietary supplementations led to an increase of these parameters (p<0.05 for the caecal tissue versus BaF group). It is worth noting that glycemia decreased in all BaF groups but the p value was significant only for BaF-INU versus CT group. Serum triglycerides, free fatty acids and glycerol as well as hepatic content of lipids were neither modified by the BaF transplantation nor by dietary treatments (Table 1).

### POS supplementation counteracts the expression of genes controlling β-oxidation in the adipose tissue

**Fig 5:**
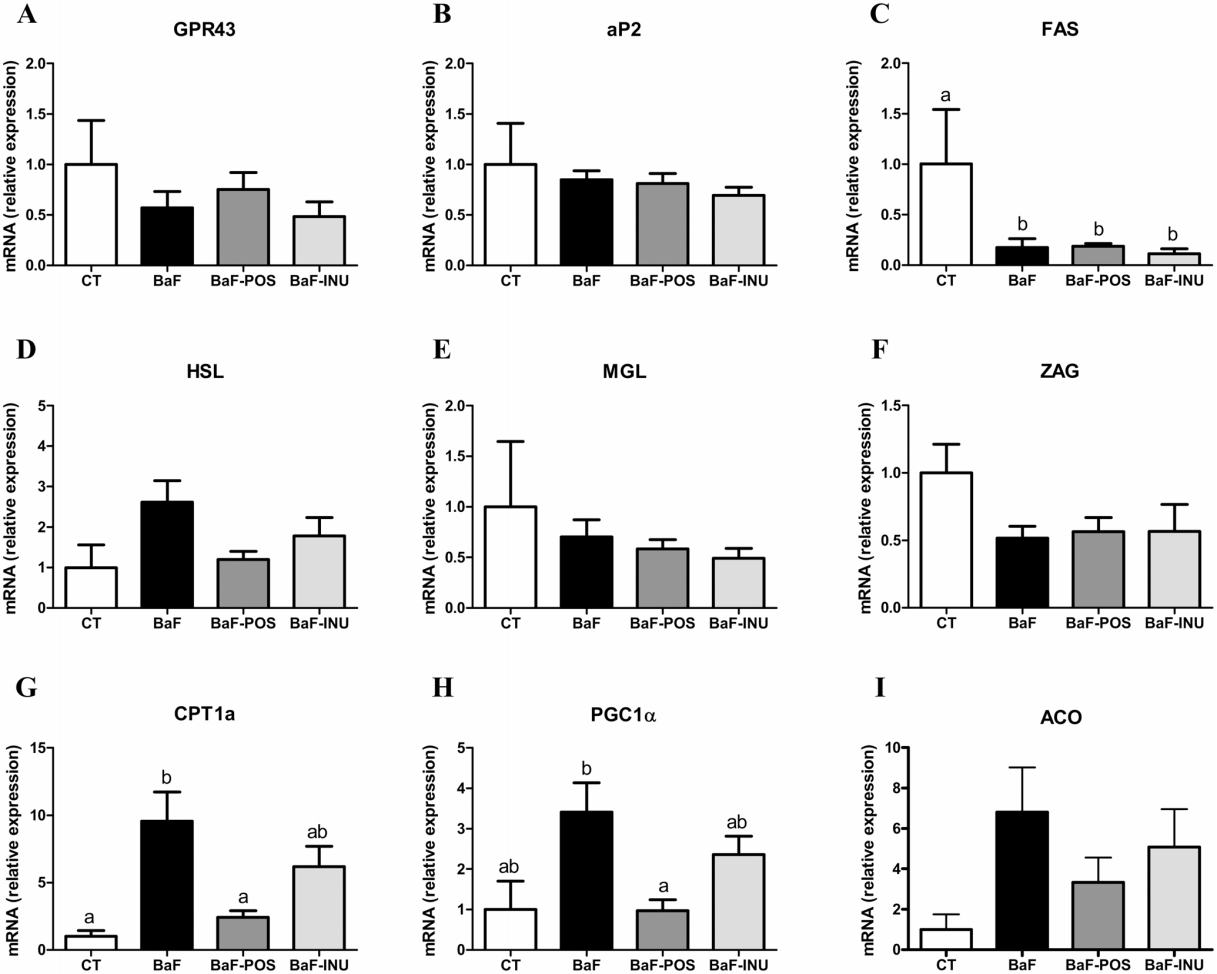
Expression of genes involved in subcutaneous adipose tissue metabolism. GPR43, G protein-coupled receptor 43 (A). aP2, adipocyte fatty acid binding protein (B). FAS, fatty acid synthase (C). HSL, hormone-sensitive lipase (D). MGL, monoglycerol lipase (E). ZAG, zinc-a2 glycoprotein (F). CPT1a, carnitine palmitoyltransferase 1a (G). PGC1α, PPARγ coactivator 1 alpha (H). ACO, acyl-CoA oxidase (I). Data with different superscript letters are significantly different.

We analyzed several genes controlling fatty acid synthesis or fatty acid oxidation in the subcutaneous adipose tissue (Fig 5). We observed a lower expression of the fatty acid synthase (FAS) in BaF groups versus CT group. This gene, controlled by SREBP1c, as well as those reflecting PPARγ activation (i.e., adipocyte Protein 2 (aP2), and G protein-coupled receptor 43 (GPR43)) were not affected by dietary treatments (Fig 5A–5C). Interestingly, although not reaching statistical significance, POS administration reduced the expression of the hormone sensitive lipase (HSL) induced after BaF injection without affecting the expression of the monoglycerol lipase (MGL) and the fat catabolic factor zinc-a2 glycoprotein (ZAG) (Fig 5D–5F). Importantly, PPARα dependent genes controlling β-oxidation (carnitine palmitoyltransferase 1a (CPT1a), PPARγ coactivator 1 alpha (PGC1α) and acyl-CoA oxidase (ACO)) were induced in BaF-transplanted mice and blunted in POS treated mice (Fig 5G–5I). The analysis of fatty acid profile in this adipose tissue showed a decrease in saturated fat in BaF groups, mostly palmitate (C16:0), which is in accordance with a lower *de novo* lipogenesis, and a decrease in oleate (C18:1) (S5 Table). A bacterial metabolite of polyunsaturated fatty acids, namely the conjugated linoleic acid *trans*-10, *cis*-12-18:2, was decreased in BaF samples, and the level was restored after INU and POS treatment.

### INU decreases cancer cell spreading in the liver, whereas both POS and INU treatments have no notable effect on tissue inflammation

Bcr-Abl is a chimeric protein, solely and constitutively expressed in BaF3 cells. We have previously shown that Bcr-ABl is a valuable marker of BaF3 cell presence [20]. In the liver, BaF3 cell tissue infiltration was associated with an increased expression of proinflammatory cytokines (IL1β, TNFα) and chemokine (MCP-1), and of a transmembrane glycoprotein (CD68) which is highly expressed by recruited monocytes and tissue macrophages (S6 Table). INU reduced the hepatic expression of Bcr-Abl in BaF-transplanted mice, reflecting a lower tumor cell infiltration in the hepatic parenchyma. This effect was accompanied by a decreased expression of MCP-1. POS had no significant effect on those parameters, neither in the liver nor in the subcutaneous adipose tissue (S6 Table).

## Discussion

Recent data from our group and others have associated microbial dysbiosis to undernutrition and cachexia [13, 20, 26, 27]. Probiotics and prebiotics have been proposed as innovative approaches to improve therapeutic outcome in undernourished individuals [28]. There is thus a clear need to test the effectiveness of potential probiotics and prebiotics in this context [28]. Recovery of prebiotics from plant biomass sources has gained much attention over the last few years. Pectin-derived oligosaccharides (POS) can be produced from sugar beet pulp, which is a large volume byproduct of the beet sugar industry. POS have been described as promising prebiotic candidates due to their interesting fermentation properties, demonstrated so far only *in vitro* [10, 12, 29]. The bifidogenic effect of POS has previously been reported *in vitro*, however with some controversy, probably because some *Bifidobacterium* strains are not able to metabolize galacturonan oligosaccharides [10, 12, 29]. In the present study, we assessed for the first time the effect of sugar beet POS on gut microbiota composition and function *in vivo*, in a mouse model of leukemia. This model is characterized at its final stage by a loss of fat mass, muscle atrophy, anorexia and inflammation [13]. The effects of POS supplementation on gut microbiota and on host health were compared to those observed with the better known prebiotics, inulin (INU). Here, we show that POS are able to stimulate *in vivo* the growth of bifidobacteria—considered as the classical “microbial” signature of prebiotics—in a more effective way than INU (6-fold *versus* 2-fold). Importantly, the use of three independent 16S rRNA gene-based techniques revealed that the relative abundance of one microbe, *Bacteroides dorei*, was increased upon POS supplementation from 1 to 23% of total bacteria. This change likely explains the reduced microbial diversity and richness observed with the POS treatment and is in accordance with previous results obtained through *in vitro* fermentation of POS [12, 29]. *B*. *dorei* is a recently described species of the genus *Bacteroides*, which appears to be closely related to *Bacteroides vulgatus* ATCC 8482(T) [30]. Its impact on host physiology has not been investigated yet. Interestingly, recent studies have found a link between the abundance of the *Bacteroides* genus or some *Bacteroides* species and adiposity [31–33].

*Bifidobacterium* and *Bacteroides* species are both able to produce acetate from hexoses *in vivo* [34]. In accordance with an *in vitro* study testing the fermentability of sugar beet pulp-derived oligosaccharides, acetate was increased in the caecal content of POS-fed mice [29]. This load of SCFA produced upon fermentation could contribute to energy sparing and modulation of host metabolism, which are particularly interesting in the context of cancer cachexia. First, acetate can be used in the adipocytes and the hepatocytes as a substrate for cytosolic acetylCoA synthesis, thereby providing an alternative source to glucose for *de novo* lipogenesis and fat storage. This could be particularly interesting since glycemia is decreased during cancer development. However, here, the glycemia was not significantly modified by POS supplementation. Second, acetate and propionate could contribute to inhibit lipolysis in the adipose tissue by interacting with the GPR43 receptor [35]. However, since SCFA portal levels did not increase with POS, it is likely that the effect of those metabolites in the POS group remains confined to the gut.

The loss of fat mass is a key feature of cancer cachexia [19]. Genetic inhibition of lipolysis allowed to maintain adipose and muscle mass in mouse models of cancer cachexia [36]. But the factors promoting lipolysis, and thereby loss of adipose tissue, are poorly understood [19, 37]. Increased production of lipolytic factors from adipose tissue such as tumor necrosis factor alpha (TNFα) or zinc-a2 glycoprotein (ZAG) could explain the increased lipolysis in cancer cachexia [38]. However, we did not observe any effect of POS on the expression of these lipolytic factors in adipose tissue. One study attributed adipocyte lipolysis to an enhanced expression and function of adipocyte hormone-sensitive lipase (HSL) and, therefore, it was suggested that the selective inhibition of this enzyme may prevent fat loss in cancer patients [36, 39]. In our study, we observed that POS administration counteracted the induction of HSL expression occurring in BaF mice. We also observed in BaF mice a higher expression of genes involved in fatty acid oxidation dependent on PPARα activity. Interestingly, in contrast to INU, POS administration blunted adipose tissue fatty acid catabolism, providing another explanation to the “adipose tissue sparing” effect exerted by the POS supplementation. Conversely, the lipogenic pathway controlled by SREBP1c analyzed through the fatty acid synthase (FAS) expression was reduced in BaF mice without any effect of dietary supplementations (INU or POS).

Finally, we describe for the first time that cancer development leads to a decrease in the amount of conjugated linoleic acid *trans*-10,*cis*-12-18:2, which is restored by both INU and POS. This metabolite is produced from polyunsaturated fatty acid (linoleic acid) upon fermentation by specific bacteria, such as *Bifidobacterium* spp., or *Roseburia* spp., which are both increased upon prebiotic treatments in BaF mice [40]. The potential contribution of this fatty acid metabolite on adipose tissue gene expression and metabolism could be interesting to evaluate, in order to propose other bacterial functions than SCFA production that could be attributed to the modulation of adiposity. We show that POS, and to a lesser extent INU, is able to delay the fall of the caloric intake (anorexia) observed at the late stages of cancer progression. This effect could also contribute to a better maintenance of adiposity, with, as consequence, a down regulation of the expression of genes involved in fatty acid oxidation (reduced CPT1a and PGC1α expression in adipose tissue).

Injection of BaF3 cells results in an accumulation of BaF3 cells in the liver, leading to an increase of its weight, as previously described [20, 41, 42]. INU reduced cancer cell infiltration into the liver, confirming our previous results obtained with fructo-oligosaccharides, another ITF [20]. In the present study, the reduced cancer cell accumulation in the liver was not accompanied by a decrease in liver weight. In fact, the same liver weight was in accordance with the observation that triglyceride, cholesterol and protein content was not modified by INU treatment (data not shown). This lower cancer cell invasion due to INU was accompanied by a lower hepatic expression of MCP-1. So far, we do not know if this decrease in MCP-1 could contribute, or would rather be the consequence of a decreased hepatic accumulation of BaF3 cells upon INU treatment. We propose propionate and butyrate as mediators of the effect of INU on cancer cell proliferation, because we have previously shown that these 2 SCFAs are able to decrease BaF3 cell proliferation *in vitro* [20] and their levels were increased by two fold in the portal blood of INU-treated mice.

In conclusion, for the first time, this study brings evidence that POS supplementation modulates the gut microbiota *in vivo*. Indeed, POS significantly increases *Bifidobacterium* spp., *Roseburia* spp. and *Bacteroides* spp. in the caecal content. POS leads to a drastic increase of *B*. *dorei*, which could be an interesting bacterial target in the control of host energy metabolism, as shown here in cancer cachexia. By inducing a metabolic shift in the adipose tissue, POS supplementation could contribute to fat mass sparing. In addition, our data support a role for portal SCFA as mediators of INU in the control of cancer cell proliferation. Taken together, this study demonstrates that non digestible carbohydrates with prebiotic properties may constitute a new nutritional strategy to modulate gut microbiota with positive consequences for cancer and associated cachexia.

## Supporting Information

S1 Fig PCR-DGGE profiles of caecal content samples for total bacteria and Bacteroides/Prevotella group. (TIFF)

S1 File Materials and methods: 454-pyrosequencing, Denaturing Gradient Gel Electrophoresis (DGGE) and qPCR analysis of the 16S rRNA gene. (PDF)

S1 Table Sugar compositions of pectic oligosaccharides (POS) prepared from beet pulp. (DOCX)

S2 Table Primer sequences used for q-PCR and PCR-DGGE. (DOC)

S3 Table Abundance of bacteria taxa, expressed in percentage, that are impacted by the dietary treatment and/or the injection of leukemic cells, as determined by pyrosequencing of 16sRNA tags. (DOCX)

S4 Table Identification of species found in the DGGE-profiles. (DOC)

S5 Table Fatty acid profile in subcutaneous adipose tissue. (DOCX)

S6 Table Expression of leukemia- and inflammation-related markers in tissues. (DOCX)
