# Supplementary material for: Non Digestible Oligosaccharides Modulate the Gut Microbiota to Control the Development of Leukemia and Associated Cachexia in Mice
Source: PLoS One. 2015 Jun 22;10(6):e0131009. doi: 10.1371/journal.pone.0131009 (PMC4476728; doi:10.1371/journal.pone.0131009)
Supplement: S1 File — (PDF) [file pone.0131009.s002.pdf]

Supporting Information Material and Method. 454-pyrosequencing, Denaturing Gradient Gel Electrophoresis (DGGE) and qPCR analysis of the 16S rRNA gene.

454-pyrosequencing: The primers E9-29 and E514-430 encompassing hypervariable regions V1-V3 specific for bacteria (1), were selected for their theoretical ability to generate the least bias of amplification among the various bacterial phyla (2). The oligonucleotide design included 454 Life Sciences A or B sequencing titanium adapters (Roche Diagnostics, Vilvoorde, Belgium) and multiplex identifiers (MIDs) fused to the 5' end of each primer. The amplification mix contained 5 U of FastStart high fidelity polymerase (Roche Diagnostics, Vilvoorde, Belgium), 1x enzyme reaction buffer, 200 µM dNTPs (Eurogentec, Liège, Belgium), 0.2 µM of each primer and 100 ng of genomic DNA in a volume of 100 µl. Thermocycling conditions consisted of a denaturation at 94°C for 15 min followed by 25 cycles of 94°C for 40 s, 56°C for 40 s, 72°C for 1 min and a final elongation step of 7 min at 72°C. These amplifications were performed on an Ep Master system gradient apparatus (Eppendorf, Hamburg, Germany). The PCR products were run on a 1% agarose gel electrophoresis and the DNA fragments were plugged out and purified using the SV PCR purification kit (Promega Benelux, Leiden, the Netherlands). The quality and quantity of the products were assessed with a Picogreen dsDNA quantitation assay (Isogen, St-Pieters-Leeuw, Belgium). All libraries were run in the same titanium pyrosequencing reaction using Roche MIDs. All amplicons were sequenced using the Roche GS-Junior Genome Sequencer instrument (Roche, Vilvoorde, Belgium). An average of 5135 reads/sample was obtained.

The 16S rRNA gene reads were processed with the MOTHUR package (3). The quality of all sequence reads were denoised using the Pyronoise algorithm implemented in MOTHUR and filtered with the following criteria: minimal length of 425 bp, an exact match to the barcode and 1 mismatch allowed to the proximal primer. The sequences were checked for the presence of chimeric amplifications using Uchime (4). The resultant read sets were compared to a reference

dataset of aligned sequences of the corresponding region derived from the SILVA database of full-length rRNA gene sequences (http://www.arb-silva.de/) implemented in MOTHUR (5). The final reads were clustered into operational taxonomic units (OTUs) using the nearest neighbor algorithm using MOTHUR with a 0.03 distance unit cutoff. A taxonomic identity was attributed to each OTU by comparison with the SILVA database (80% homogeneity cutoff). As MOTHUR is not dedicated to the taxonomic assignment beyond the genus level, all unique sequences for each OTU were compared to the SILVA dataset 111 using BLASTN algorithm (6). For each OTU, a consensus detailed taxonomic identification has been given based upon the identity (less than 1% of mismatch with the aligned sequence) and the metadata associated with the best hit (validated bacterial species or not). Subsampled datasets at 3000 reads/sample were obtained and used to evaluate richness and microbial diversity using MOTHUR. Microbial biodiversity (non parametric (NP) Shannon diversity index (7) and richness estimation (Jackknife estimate (8)) were calculated. The NP Shannon index gives an estimated index value for diversity and is used when undetected species are present in the sample. The Jackknife estimator is used to estimate the unbiased species (OTUs in this case) richness in the sample. We used ANOVA, including a false-discovery correction according to the Benjamini and Hochberg procedure, to highlight statistical differences in the relative abundance of bacterial population between groups. Post-hoc Tukey-Kramer test were performed to assess differences between each group pair. To do this, we used the STAMP software which has been developed for metagenomic data analysis (9). The differences were considered significant for a corrected p-value of less than 0.05. The sequences used for analysis can be found in the SRA-genbank database, with the following accession numbers: SAMN03580736 to SAMN03580767.

Denaturing Gradient Gel Electrophoresis (DGGE): DGGE was performed on total bacteria and *Bacteroides/Prevotella* group. All amplification products were checked by electrophoresis on a 1.5 % agarose gel. Total bacteria amplicons were generated with primers targeting the V3 region of bacterial 16S rRNA gene (338F with a GC clamp of 40 bp and 518R) (10) whereas a nested PCR was performed to obtain *Bacteroides-Prevotella* amplicons: *Bacteroides-Prevotella* group-specific primers FD1/RBactPr was first used, followed by a second PCR amplification with the universal primers from the V3 region (338F-GC and 518R) (11). The sequences of primers are reported in Supplemental Table 2. The PCR products were analyzed on a 40% to 60% DGGE gel for total bacteria and for *Bacteroides*, according to manufacturer's instructions (Ingeny phorU-2, Ingeny International, The Netherlands). The gel was stained with SYBR Gold (Invitrogen) for 30 min and visualized by UV radiation. The DGGE fingerprints obtained were analyzed using Gel ComparII 6.0 software (Applied Maths Sint- Martens-Latem, Belgium) using Dice coefficients and the unweighted-pair group method with averages (UPGMA) for the generation of dendrograms. Specific bands were excised, DNA was re-amplified using 338F and 518R primers and the PCR products were subjected to sequencing using the services of Macrogen (Amsterdam, The Netherlands). The sequences were compared with those available in the GenBank database using NCBI BLAST.

Quantitative polymerase chain reaction (qPCR): q-PCR was performed with a StepOnePlus Real-Time PCR System and software (Applied Biosystems, Den Ijssel, The Netherlands) using Mesa Fast qPCR™ (Eurogentec, Seraing, Belgium) for detection. The primers are detailed in Supplemental Table 2. The cycle threshold of each sample was compared with a standard curve made by diluting genomic DNA isolated from a pure culture of a type strain (BCCM/LMG, Ghent, Belgium; DSMZ, Braunshweig, Germany). Prior to DNA isolation, the cell counts of

these cultures were determined by culture (CFU) or flow cytometry (bacterial cell). For *Bacteroides dorei/vulgatus*, standard curve consists in serial dilutions of a sample that showed a strong band intensity on the *Bacteroides-Prevotella* DGGE, and results are expressed as arbitrary units/g of caecal content.

References

1. Brosius J, Dull TJ, Sleeter DD, Noller HF. Gene organization and primary structure of a ribosomal RNA operon from Escherichia coli. J Mol Biol. 1981;148(2):107-27.
2. Wang Y, Qian PY. Conservative fragments in bacterial 16S rRNA genes and primer design for 16S ribosomal DNA amplicons in metagenomic studies. PLoS One. 2009;4(10):e7401.
3. Schloss PD, Westcott SL, Ryabin T, Hall JR, Hartmann M, Hollister EB, et al. Introducing mothur: open-source, platform-independent, community-supported software for describing and comparing microbial communities. Appl Environ Microbiol. 2009;75(23):7537-41.
4. Edgar RC, Haas BJ, Clemente JC, Quince C, Knight R. UCHIME improves sensitivity and speed of chimera detection. Bioinformatics. 2011;27(16):2194-200.
5. Pruesse E, Quast C, Knittel K, Fuchs BM, Ludwig W, Peplies J, et al. SILVA: a comprehensive online resource for quality checked and aligned ribosomal RNA sequence data compatible with ARB. Nucleic Acids Res. 2007;35(21):7188-96.
6. Altschul SF, Gish W, Miller W, Myers EW, Lipman DJ. Basic local alignment search tool. J Mol Biol. 1990;215(3):403-10.
7. Chao A, Shen T-J. Nonparametric estimation of Shannon’s index of diversity when there are unseen species in sample. Environmental and Ecological Statistics. 2003;10(4):429-43.
8. Burnham KP, Overton WS. Robust Estimation of Population Size When Capture Probabilities Vary Among Animals. Ecology. 1979;60(5):927-36.
9. Parks DH, Beiko RG. Identifying biologically relevant differences between metagenomic communities. Bioinformatics. 2010;26(6):715-21.
10. Ovreas L, Forney L, Daae FL, Torsvik V. Distribution of bacterioplankton in meromictic Lake Saelenvannet, as determined by denaturing gradient gel electrophoresis of PCR-amplified gene fragments coding for 16S rRNA. Appl Environ Microbiol. 1997;63(9):3367-73.
11. Wood J, Scott KP, Avgustin G, Newbold CJ, Flint HJ. Estimation of the relative abundance of different Bacteroides and Prevotella ribotypes in gut samples by restriction enzyme profiling of PCR-amplified 16S rRNA gene sequences. Appl Environ Microbiol. 1998;64(10):3683-9.
