# Supplementary material for: Non Digestible Oligosaccharides Modulate the Gut Microbiota to Control the Development of Leukemia and Associated Cachexia in Mice
Source: PLoS One. 2015 Jun 22;10(6):e0131009. doi: 10.1371/journal.pone.0131009 (PMC4476728; doi:10.1371/journal.pone.0131009)
Supplement: S1 Table — (DOCX) [file pone.0131009.s003.docx]

**Online Supporting Material**

**Supplemental Table 1** Sugar compositions of pectic oligosaccharides (POS) prepared from beet pulp

| Saccharides (% dry matter) | Total | Free |
| --- | --- | --- |
| Rhamnose | 1.95 | 0.00 |
| Arabinose | 3.85 | 1.03 |
| Xylose | 0.00 | 0.00 |
| Mannose | 6.65 | 0.63 |
| Glucose | 14.32 | 1.72 |
| Galactose | 5.90 | 0.19 |
| Galacturonic Acid | 43.78 | 2.12 |
| Sucrose | n.d. | 0.03 |
| TOTAL saccharides (% dry matter) | 80.04 | |
| degree of acetylation (%GalA) | 13.53 | |
| degree of methylation (%GalA) | 13.01 | |
| degree of polymerization (GalA unit) | 22 | |

**GalA, galacturonic acid**

**POS preparation and characterization**

Pectin was extracted from sugar beet pulp by acid treatment according to the protocol of Combo *et al*.,([1](#_ENREF_1)) slightly modified and adapted at a pilot scale. A solid-liquid ratio of 1:29 (w/v) was suspended in an aqueous solution adjusted to pH 1.25 with concentrated HCl, heated to 80°C and stirred for 2 hours. The macerate was cooled and ultrafiltered (MWcutoff : 50 kDa). The extract was further purified by ethanol precipitation. POS extract was prepared enzymatically by treating the pectin extract with Rapidase® Smart (DSM Food, The Netherlands). Pectin was diluted in an aqueous solution to a final concentration of 1% (w/v) and the pH was adjusted to 5 (optimal working range of the enzyme). Temperature was set to 50°C. A volume of 24 ml of Rapidase Smart was added to 300 g of substrate. At the end of hydrolysis (15 minutes), the sample was heated to 100°C to inactivate the enzyme, concentrated under reduced pressure and finally freeze-dried.

The molecular weight distribution of the sample was analyzed by HPSEC (Water 2690 HPLC system) equipped with a refractive index detector. Neutral sugars were analyzed by gas chromatography after enzymatic hydrolysis and conversion to alditol acetates. Galacturonic acid was determined by a high performance anion-exchange chromatography with pulsed amperometric detection. Methoxy and acetyl groups were released from POS by saponification and quantified by high performance liquid chromatography, as described in Combo *et al.*.([1](#_ENREF_1))

1. Combo AM, Aguedo M, Quievy N*.* Characterization of sugar beet pectic-derived oligosaccharides obtained by enzymatic hydrolysis. Int J Biol Macromol 2013;52:148-56.
