# Supplementary material for: Non Digestible Oligosaccharides Modulate the Gut Microbiota to Control the Development of Leukemia and Associated Cachexia in Mice
Source: PLoS One. 2015 Jun 22;10(6):e0131009. doi: 10.1371/journal.pone.0131009 (PMC4476728; doi:10.1371/journal.pone.0131009)
Supplement: S2 Table — (DOC) [file pone.0131009.s004.doc]

**Online Supporting Material**

**Supplemental Table 2** Primer sequences used for q -PCR and PCR -DGGE.

|  | **Primer Forward** | **Primer Reverse** |
| --- | --- | --- |
| **qPCR** |  |  |
| RPL19 (housekeeping gene) | GAAGGTCAAAGGGAATGTGTTCA | CCTTGTCTGCCTTCAGCTTGT |
| CPT1a | AGACCGTGAGGAACTCAAACCTAT | TGAAGAGTCGCTCCCACT |
| FAS | TTCCAAGACGAAAATGATGC | AATTGTGGGATCAGGAGAGC |
| GPR43 | GGGATCTGGGTCACATGCTTAT | ATGTCAGACAGACGGGTACCAA |
| aP2 | GATGCCTTTGTGGGAACCTG | GCCATGCCTGCCACTTTC |
| PGC1α | AGCCGTGACCACTGACAACGAG | GCTGCATGGTTCTGAGTGCTAAG |
| HSL | GCTAGCCAGGCTCATCTCCT | GTTCTTGAGGTAGGGCTCGT |
| ACO | CTATGGGATCAGCCAGAAAGG | AGTCAAAGGCATCCACCAAAG |
| ZAG | gtggactgttctcacgctcaag | tccgtccttcgcttcatagg |
| MGL | cactgctctgtcttgtgtaggttg | tcgttgtgcctttattagtgcatc |
| MCP1 | GCAGTTAACGCCCCACTCA | CCCAGCCTACTCATTGGGATCA |
| CD68 | CTTCCCACAGGCAGCACAG | AATGATGAGAGGCAGCAAGAGG |
| TNFα | AGCCCCCAGTCTGTATCCTT | GGTCACTGTCCCAGCATCTT |
| IL1β | TCGCTCAGGGTCACAAGAAA | CATCAGAGGCAAGGAGGAAAAC |
| Bacteria Universal  338F and 518R | ACTCCTACGGGAGGCAGCAG | ATTACCGCGGCTGCTGG |
| *Bifidobacterium* | GATTCTGGCTCAGGATGAACGC | CTGATAGGACGCGACCCCAT |
| *Bifidobacterium animalis* | ACCAACCTGCCCTGTGCACCG | CCATCACCCCGCCAACAAGCT |
| *Bacteroides* – *Prevotella* | GAGAGGAAGGTCCCCCAC | CGCTACTTGGCTGGTTCAG |
| *Lactobacillus* | AGCAGTAGGGAATCTTCCA | CACCGCTACACATGGAG |
| *Roseburia* | AAGCGACGATCAGTAGCCGA | TTCTTCTTCCCTGCTGATAGAG |
| *Bacteroides dorei/vulgatus* | TCAACGATGGATAGGGGTTC | TGCATACCCGACTTTATTCCTT |
| *Akkermansia muciniphila* | CAGCACGTGAAGGTGGGGAC | CCTTGCGGTTGGCTTCAGAT |
| **DGGE** |  |  |
| Bacteria Universal  338F-GC and 518R | GC-clamp + ACTCCTACGGGAGGCAGCAG | ATTACCGCGGCTGCTGG |
| *Bacteroides*  FD1 and RBacPre | AGAGTTTGATCCTGGCTCAG | TCACCGTTGCCGGCGTACTC |

GC-clamp: 5’-CGCCCGCCGCGCGCGGCGGGCGGGGCGGGGGCACGGGGG-3´

RPL19: Ribosomal protein L19; CPT1a: Carnitine palmitoyl transferase 1 a; FAS: Fatty acid synthase; GPR43, G-protein-coupled receptor; aP2, adipocyte Protein 2; PGC1α: Peroxisome proliferator-activated receptor gamma coactivator 1-alpha; HSL, hormone-sensitive lipase; ACO, acyl-CoA oxidase; ZAG, zinc-a2 glycoprotein; MGL, monoglycerol lipase; MCP1, monocyte chemoattractant protein 1; TNFα, tumor necrosis factor alpha; IL1β, interleukin 1 beta.
