# Supplementary material for: Non Digestible Oligosaccharides Modulate the Gut Microbiota to Control the Development of Leukemia and Associated Cachexia in Mice
Source: PLoS One. 2015 Jun 22;10(6):e0131009. doi: 10.1371/journal.pone.0131009 (PMC4476728; doi:10.1371/journal.pone.0131009)
Supplement: S3 Table — (DOCX) [file pone.0131009.s005.docx]

**Online Supporting Material**

**Supplemental Table 3** Abundance of bacteria taxa, expressed in percentage, that are impacted by the dietary treatment and/or the injection of leukemic cells, as determined by pyrosequencing of 16sRNA tags.

1a. At the phylum level

| Phylum | Corrected p-value | CT  mean | CT  SD | BaF  mean | BaF  SD | BaF-POS  mean | BaF-POS  SD | BaF-INU  mean | BaF-INU  SD |
| --- | --- | --- | --- | --- | --- | --- | --- | --- | --- |
| Firmicutes | 4,376E-05 | 54,230a | 8,216 | 48,929ac | 9,841 | 27,936b | 7,923 | 40,970c | 4,442 |
| Bacteroidetes | 7,412E-04 | 39,654a | 8,516 | 37,148a | 7,346 | 58,262b | 7,661 | 44,327a | 9,617 |
| Candidate_division_TM7 | 0,035 | 0,094a | 0,079 | 0,305a | 0,264 | 0,003b | 0,009 | 0,130a | 0,174 |

1b. At the family level

| Family | Corrected p-value | CT  mean | CT  SD | BaF  mean | BaF  SD | BaF-POS  mean | BaF-POS  SD | BaF-INU  mean | BaF-INU  SD |
| --- | --- | --- | --- | --- | --- | --- | --- | --- | --- |
| Prevotellaceae | 3,097E-09 | 0,473a | 0,471 | 0,228a | 0,260 | 7,030b | 2,649 | 0,101a | 0,232 |
| Desulfovibrionaceae | 1,699E-07 | 2,122a | 0,629 | 1,084b | 0,498 | 0,322c | 0,104 | 0,471c | 0,162 |
| Anaeroplasmataceae | 8,787E-06 | 0,000a | 0,000 | 0,000a | 0,000 | 0,438b | 0,266 | 0,014a | 0,026 |
| Bacteroidaceae | 1,326E-05 | 3,623a | 3,120 | 2,566a | 1,248 | 24,122b | 8,635 | 9,135a | 8,651 |
| Rikenellaceae | 0,003 | 8,333a | 1,587 | 5,775ab | 2,912 | 6,806a | 2,760 | 2,851b | 0,701 |
| S24-7 | 0,002 | 7,959a | 2,511 | 7,811a | 1,673 | 5,138b | 1,480 | 9,249a | 1,460 |
| Ruminococcaceae | 0,005 | 18,149a | 3,579 | 15,092a | 3,997 | 9,822b | 2,810 | 14,428ab | 3,661 |

1c. At the genus level

| Genus | Corrected p-value | CT  mean | CT  SD | BaF  mean | BaF  SD | BaF-POS  mean | BaF-POS  SD | BaF-INU  mean | BaF-INU  SD |
| --- | --- | --- | --- | --- | --- | --- | --- | --- | --- |
| Anaeroplasma | 1,17E-05 | 0,000a | 0,000 | 0,000a | 0,000 | 0,438b | 0,266 | 0,014a | 0,026 |
| Anaerostipes | 1,70E-05 | 0,099a | 0,051 | 0,005b | 0,014 | 0,012b | 0,025 | 0,000b | 0,000 |
| Bacteroides | 1,77E-05 | 3,623a | 3,120 | 2,566a | 1,248 | 24,122b | 8,635 | 9,135a | 8,651 |
| Alistipes | 0,004 | 8,333a | 1,587 | 5,775ab | 2,912 | 6,806a | 2,760 | 2,851b | 0,701 |
| Oscillospira | 0,004 | 0,977a | 0,282 | 0,731a | 0,359 | 0,280b | 0,165 | 0,900a | 0,360 |
| Ruminococcaceae_uncultured | 0,010 | 2,584a | 1,348 | 4,574a | 1,359 | 6,415ab | 3,318 | 8,789b | 3,600 |
| Oscillibacter | 0,018 | 0,871a | 0,667 | 0,469ab | 0,274 | 0,153b | 0,111 | 0,287b | 0,155 |
| Hydrogenoanaerobacterium | 0,022 | 0,097a | 0,062 | 0,058ab | 0,075 | 0,019b | 0,025 | 0,002b | 0,006 |
| Coprobacillus | 0,023 | 0,000a | 0,000 | 0,000a | 0,000 | 0,000a | 0,000 | 0,054b | 0,064 |

1d. At the species level (OTUs)

| Mothur_hit | Blast_hit | Corrected p-value | CT  mean | CT  SD | BaF  mean | BaF  SD | BaF-POS  mean | BaF-POS  SD | BaF-INU  mean | BaF-INU  SD |
| --- | --- | --- | --- | --- | --- | --- | --- | --- | --- | --- |
| **Prevotellaceae_unclassified** | **Prevotellaceae_EF603170** | **4,478E-08** | **0,293a** | **0,368** | **0,088a** | **0,066** | **3,411b** | **1,228** | **0,042a** | **0,092** |
| Lachnospiraceae_unclassified | Lachnospiraceae_EF098072/AF157053 | 4,553E-08 | 0,005 | 0,011 | 0,021 | 0,027 | 0,031 | 0,026 | 2,116 | 0,832 |
| **Bacteroidaceae_Bacteroides** | **Bacteroides_HQ788586** | **6,542E-08** | **1,275a** | **1,891** | **1,138a** | **1,027** | **22,919b** | **8,573** | **1,144a** | **1,751** |
| Ruminococcaceae_Incertae_Sedis | Ruminococcaceae_JQ084814 | 1,678E-07 | 1,351 | 0,207 | 0,519 | 0,340 | 0,117 | 0,142 | 0,239 | 0,179 |
| Lachnospiraceae_unclassified | Lachnospiraceae_JQ084349 | 7,631E-07 | 0,635 | 0,200 | 0,137 | 0,194 | 0,014 | 0,026 | 0,002 | 0,005 |
| Desulfovibrionaceae_unclassified | Bilophila_JQ085186 | 1,645E-06 | 2,122 | 0,629 | 1,084 | 0,498 | 0,322 | 0,104 | 0,471 | 0,162 |
| Lachnospiraceae_unclassified | Lachnospiraceae_EU510904 | 2,331E-06 | 0,447 | 0,149 | 0,236 | 0,150 | 0,012 | 0,014 | 0,025 | 0,021 |
| Ruminococcaceae_unclassified | Anaerotruncus_JQ084752 | 2,904E-06 | 0,000 | 0,000 | 0,019 | 0,025 | 0,605 | 0,284 | 0,059 | 0,090 |
| Clostridiales_unclassified | Lachnospiraceae_DQ015429 | 3,219E-06 | 1,102 | 0,418 | 0,927 | 0,489 | 0,000 | 0,000 | 0,005 | 0,009 |
| Lachnospiraceae_unclassified | Lachnospiraceae_EU457504 | 5,586E-06 | 0,446 | 0,241 | 0,042 | 0,044 | 0,002 | 0,006 | 0,009 | 0,013 |
| Clostridiales_unclassified | Lachnospiraceae_AB606296/EU006423 | 5,911E-06 | 0,000 | 0,000 | 0,000 | 0,000 | 0,000 | 0,000 | 1,035 | 0,557 |
| **Rikenellaceae_Alistipes** | **Alistipes_DQ815965/EF603688** | **1,144E-05** | **2,420a** | **0,739** | **1,326b** | **0,704** | **0,151c** | **0,085** | **1,418b** | **0,366** |
| Clostridiales_unclassified | Lachnospiraceae_EU508687/EU508758 | 1,789E-05 | 3,829 | 1,395 | 2,937 | 1,931 | 0,000 | 0,000 | 0,008 | 0,021 |
| **Prevotellaceae_unclassified** | **S24-7_AM265451** | **2,592E-05** | **0,180a** | **0,136** | **0,140a** | **0,244** | **3,626b** | **2,056** | **0,059a** | **0,141** |
| **Rikenellaceae_Alistipes** | **Alistipes_JQ085082** | **3,941E-05** | **3,214a** | **0,698** | **2,943a** | **1,801** | **0,087b** | **0,092** | **0,507b** | **0,524** |
| Lachnospiraceae_unclassified | Lachnospiraceae_JQ084500 | 4,005E-05 | 0,070 | 0,075 | 0,043 | 0,035 | 0,306 | 0,313 | 1,722 | 0,914 |
| Anaeroplasmataceae_Anaeroplasma | Anaeroplasma_JQ084129 | 4,030E-05 | 0,000 | 0,000 | 0,000 | 0,000 | 0,438 | 0,266 | 0,014 | 0,026 |
| Lachnospiraceae_Anaerostipes | Lachnospiraceae_HM844169 | 4,126E-05 | 0,099 | 0,051 | 0,005 | 0,014 | 0,012 | 0,025 | 0,000 | 0,000 |
| **Rikenellaceae_Alistipes** | **Alistipes_JQ084893** | **4,333E-05** | **0,744a** | **0,369** | **0,743a** | **0,277** | **5,678b** | **3,066** | **0,490a** | **0,232** |
| Ruminococcaceae_unclassified | Ruminococcaceae_EF406869 | 5,803E-05 | 0,275 | 0,165 | 0,063 | 0,042 | 0,009 | 0,020 | 0,002 | 0,006 |
| Rikenellaceae_Alistipes | Alistipes_EF603418 | 6,297E-05 | 1,761 | 0,662 | 0,642 | 0,482 | 0,285 | 0,267 | 0,325 | 0,166 |
| Lachnospiraceae_unclassified | Coprococcus_EF406589 | 6,537E-05 | 0,118 | 0,068 | 0,061 | 0,024 | 0,002 | 0,006 | 0,012 | 0,012 |
| Lachnospiraceae_unclassified | Lachnospiraceae_JQ084100 | 1,381E-04 | 1,102 | 0,576 | 0,506 | 0,294 | 0,169 | 0,113 | 0,065 | 0,069 |
| Lachnospiraceae_unclassified | Lachnospiraceae_AB626945 | 1,406E-04 | 1,033 | 0,519 | 0,577 | 0,466 | 0,005 | 0,014 | 0,002 | 0,005 |
| Ruminococcaceae_unclassified | Christensenellaceae_hydrasante_2013_OTU275 | 1,802E-04 | 0,000 | 0,000 | 0,011 | 0,019 | 0,003 | 0,009 | 0,065 | 0,036 |
| Lachnospiraceae_unclassified | Lachnospiraceae_EU454464 | 1,958E-04 | 0,059 | 0,045 | 0,168 | 0,095 | 0,008 | 0,021 | 0,015 | 0,010 |
| Lachnospiraceae_unclassified | Lachnospiraceae_EU510123/EF406831 | 2,628E-04 | 0,819 | 0,404 | 0,372 | 0,327 | 0,069 | 0,128 | 0,003 | 0,009 |
| Ruminococcaceae_unclassified | Ruminococcaceae_EU456461 | 3,189E-04 | 0,542 | 0,348 | 0,887 | 0,534 | 0,056 | 0,088 | 0,008 | 0,011 |
| Ruminococcaceae_unclassified | Ruminococcaceae_EU457329 | 3,546E-04 | 2,061 | 1,361 | 2,050 | 1,065 | 0,145 | 0,123 | 0,165 | 0,105 |
| Lachnospiraceae_unclassified | Lachnospiraceae_JQ084556 | 4,566E-04 | 0,954 | 0,601 | 1,779 | 0,815 | 0,034 | 0,053 | 0,572 | 0,537 |
| Lachnospiraceae_unclassified | Lachnospiraceae_hydrasante_2013_OTU285 | 5,305E-04 | 0,146 | 0,095 | 0,024 | 0,021 | 0,008 | 0,022 | 0,011 | 0,019 |
| Ruminococcaceae_Oscillibacter | S24-7_EU508149 | 9,356E-04 | 0,179 | 0,132 | 0,022 | 0,039 | 0,006 | 0,009 | 0,002 | 0,005 |
| Lachnospiraceae_unclassified | Lachnospiraceae_EU511795 | 1,011E-03 | 0,662 | 0,375 | 0,268 | 0,311 | 0,015 | 0,025 | 0,013 | 0,013 |
| Ruminococcaceae_unclassified | Anaerotruncus_EU503579 | 1,407E-03 | 0,227 | 0,155 | 0,232 | 0,124 | 0,033 | 0,038 | 0,016 | 0,035 |
| Lachnospiraceae_unclassified | Lachnospiraceae_EU503875/HQ740280 | 2,858E-03 | 0,005 | 0,011 | 0,016 | 0,027 | 2,108 | 1,764 | 0,000 | 0,000 |
| S24-7_unclassified | S24-7_EF100075 | 2,873E-03 | 0,105 | 0,078 | 0,009 | 0,014 | 0,021 | 0,028 | 0,000 | 0,000 |
| S24-7_unclassified | S24-7_EF097809 | 3,468E-03 | 0,111 | 0,111 | 0,134 | 0,088 | 0,016 | 0,036 | 0,287 | 0,147 |
| Bacteroidetes_unclassified | S24-7_EF603042 | 3,721E-03 | 0,022 | 0,049 | 0,021 | 0,024 | 0,002 | 0,005 | 0,513 | 0,427 |
| Ruminococcaceae_uncultured | Ruminococcaceae_hydrasante_2013_OTU278 | 4,258E-03 | 0,000 | 0,000 | 0,002 | 0,005 | 0,100 | 0,085 | 0,004 | 0,011 |
| Firmicutes_unclassified | Clostridiales_JQ084309 | 4,752E-03 | 0,042 | 0,066 | 0,201 | 0,145 | 0,035 | 0,048 | 0,390 | 0,244 |
| Lachnospiraceae_unclassified | Lachnospiraceae_HM124257 | 4,848E-03 | 0,094 | 0,075 | 0,012 | 0,020 | 0,007 | 0,020 | 0,002 | 0,004 |
| Lachnospiraceae_unclassified | Lachnospiraceae_EU510407 | 5,447E-03 | 0,288 | 0,245 | 0,047 | 0,085 | 0,000 | 0,000 | 0,000 | 0,000 |
| Ruminococcaceae_unclassified | Oscillibacter_HM822404 | 5,566E-03 | 0,012 | 0,026 | 0,010 | 0,013 | 0,000 | 0,000 | 0,075 | 0,057 |
| Firmicutes_unclassified | RF9_hydrasante_2013_OTU207 | 6,140E-03 | 0,031 | 0,037 | 0,017 | 0,016 | 0,003 | 0,007 | 0,116 | 0,086 |
| Lachnospiraceae_unclassified | Lachnospiraceae_EF099644 | 6,786E-03 | 0,036 | 0,022 | 0,065 | 0,048 | 0,036 | 0,030 | 0,294 | 0,220 |
| S24-7_unclassified | S24-7_EF406789/EF604755 | 7,284E-03 | 3,485 | 1,408 | 2,842 | 0,924 | 1,834 | 0,472 | 4,165 | 1,043 |
| Ruminococcaceae_unclassified | Ruminococcaceae_EU507524 | 7,888E-03 | 0,177 | 0,082 | 0,154 | 0,148 | 0,018 | 0,039 | 0,000 | 0,000 |
| Ruminococcaceae_unclassified | Oscillibacter_EU452918 | 8,345E-03 | 0,032 | 0,049 | 0,092 | 0,047 | 0,026 | 0,038 | 0,000 | 0,000 |
| Bacteroidales_unclassified | S24-7_EU505152 | 9,087E-03 | 0,110 | 0,104 | 0,034 | 0,031 | 0,016 | 0,022 | 0,985 | 0,880 |
| Ruminococcaceae_unclassified | Ruminococcaceae_AB622833 | 9,393E-03 | 0,000 | 0,000 | 0,003 | 0,009 | 0,000 | 0,000 | 0,456 | 0,430 |
| Bacteroidetes_unclassified | S24-7_EU791032 | 1,029E-02 | 0,000 | 0,000 | 0,003 | 0,006 | 0,004 | 0,008 | 0,243 | 0,228 |
| Bacteroidaceae_Bacteroides | Bacteroides_hydrasante_2013_OTU90 | 1,109E-02 | 0,312 | 0,241 | 0,051 | 0,070 | 0,409 | 0,316 | 0,000 | 0,000 |
| Clostridiales_unclassified | Lachnospiraceae_EU508687 | 1,182E-02 | 0,022 | 0,018 | 0,008 | 0,011 | 0,000 | 0,000 | 0,000 | 0,000 |
| Ruminococcaceae_unclassified | Ruminococcaceae_EF098326 | 1,226E-02 | 0,350 | 0,264 | 0,135 | 0,130 | 0,003 | 0,007 | 0,085 | 0,071 |
| Lachnospiraceae_unclassified | Coprococcus_AB606324 | 1,289E-02 | 0,218 | 0,125 | 0,150 | 0,154 | 0,000 | 0,000 | 0,039 | 0,039 |
| Ruminococcaceae_unclassified | Ruminococcaceae_EF602939 | 1,302E-02 | 0,232 | 0,183 | 0,592 | 0,516 | 0,037 | 0,048 | 0,020 | 0,029 |
| Ruminococcaceae_Oscillospira | Ruminococcaceae_EU506367 | 1,408E-02 | 0,696 | 0,166 | 0,550 | 0,311 | 0,142 | 0,118 | 0,339 | 0,290 |
| Ruminococcaceae_Oscillibacter | Ruminococcaceae_EU509337 | 1,993E-02 | 0,061 | 0,062 | 0,005 | 0,013 | 0,000 | 0,000 | 0,002 | 0,005 |
| Lachnospiraceae_unclassified | Lachnospiraceae_EU455256 | 2,110E-02 | 0,150 | 0,138 | 0,006 | 0,009 | 0,020 | 0,044 | 0,013 | 0,015 |
| Lachnospiraceae_unclassified | Lachnospiraceae_EU508167 | 2,127E-02 | 0,170 | 0,157 | 0,038 | 0,075 | 0,000 | 0,000 | 0,000 | 0,000 |
| Ruminococcaceae_unclassified | Ruminococcaceae_JQ084614 | 2,418E-02 | 0,033 | 0,052 | 0,000 | 0,000 | 0,038 | 0,066 | 0,186 | 0,154 |
| S24-7_unclassified | S24-7_EF406773 | 2,445E-02 | 0,311 | 0,174 | 0,170 | 0,102 | 0,111 | 0,066 | 0,092 | 0,023 |
| S24-7_unclassified | S24-7_EU791121 | 2,490E-02 | 0,000 | 0,000 | 0,035 | 0,057 | 0,150 | 0,120 | 0,025 | 0,039 |
| Lachnospiraceae_unclassified | Lachnospiraceae_HM836898 | 2,498E-02 | 0,842 | 0,790 | 0,435 | 0,255 | 0,011 | 0,032 | 0,138 | 0,149 |
| Rikenellaceae_Alistipes | Alistipes_JQ084767 | 2,521E-02 | 0,116 | 0,171 | 0,005 | 0,014 | 0,225 | 0,177 | 0,000 | 0,000 |
| Ruminococcaceae_unclassified | Ruminococcaceae_EF603425 | 2,605E-02 | 0,045 | 0,047 | 0,026 | 0,020 | 0,000 | 0,000 | 0,000 | 0,000 |
| Lachnospiraceae_unclassified | Coprococcus_EU509038 | 2,740E-02 | 0,022 | 0,025 | 0,000 | 0,000 | 0,000 | 0,000 | 0,000 | 0,000 |
| Lachnospiraceae_unclassified | Lachnospiraceae_AB606268/EU511957 | 2,875E-02 | 0,197 | 0,307 | 0,155 | 0,121 | 0,529 | 0,336 | 0,817 | 0,480 |
| Lachnospiraceae_unclassified | Lachnospiraceae_EF604625 | 3,014E-02 | 0,206 | 0,155 | 0,145 | 0,167 | 0,004 | 0,012 | 0,007 | 0,015 |
| Lachnospiraceae_unclassified | Lachnospiraceae_EU454185 | 3,025E-02 | 0,064 | 0,063 | 0,011 | 0,019 | 0,000 | 0,000 | 0,007 | 0,010 |
| Lachnospiraceae_unclassified | Lachnospiraceae_HM844409 | 3,031E-02 | 0,010 | 0,022 | 0,012 | 0,016 | 0,126 | 0,109 | 0,529 | 0,514 |
| Ruminococcaceae_unclassified | Oscillibacter_hydrasante_2013_OTU113 | 3,055E-02 | 0,054 | 0,066 | 0,033 | 0,025 | 0,036 | 0,057 | 0,362 | 0,341 |
| Lachnospiraceae_unclassified | Lachnospiraceae_FJ881092 | 3,658E-02 | 0,090 | 0,082 | 0,163 | 0,148 | 0,081 | 0,080 | 0,927 | 0,889 |
| Ruminococcaceae_unclassified | Anaerotruncus_HM832302 | 3,665E-02 | 0,020 | 0,033 | 0,087 | 0,048 | 0,022 | 0,045 | 0,017 | 0,024 |
| Ruminococcaceae_Oscillibacter | Ruminococcaceae_EU455859 | 3,742E-02 | 0,000 | 0,000 | 0,008 | 0,016 | 0,000 | 0,000 | 0,072 | 0,076 |
| Bacteroidetes_unclassified | Odoribacter_JQ084406 | 3,809E-02 | 0,660 | 0,882 | 3,781 | 1,575 | 1,803 | 1,317 | 2,820 | 1,723 |
| Lachnospiraceae_unclassified | Lachnospiraceae_EU504642 | 3,810E-02 | 0,079 | 0,091 | 0,007 | 0,014 | 0,000 | 0,000 | 0,000 | 0,000 |
| Lachnospiraceae_unclassified | Lachnospiraceae_EU453875 | 3,837E-02 | 0,107 | 0,090 | 0,045 | 0,045 | 0,016 | 0,026 | 0,008 | 0,017 |
| Ruminococcaceae_unclassified | Ruminococcaceae_EF099754 | 3,882E-02 | 0,805 | 0,794 | 0,180 | 0,195 | 0,112 | 0,071 | 0,058 | 0,069 |
| Lachnospiraceae_uncultured | Clostridiales_AB606358/EU456965 | 3,897E-02 | 0,403 | 0,631 | 0,746 | 0,866 | 0,635 | 0,815 | 3,102 | 2,501 |
| Lachnospiraceae_unclassified | Lachnospiraceae_hydrasante_2013_OTU355 | 3,991E-02 | 0,061 | 0,070 | 0,006 | 0,012 | 0,000 | 0,000 | 0,000 | 0,000 |
| Ruminococcaceae_uncultured | Ruminococcaceae_EU454118 | 4,112E-02 | 0,053 | 0,096 | 0,005 | 0,010 | 0,003 | 0,007 | 0,132 | 0,114 |
| Lachnospiraceae_unclassified | Lachnospiraceae_JQ084870 | 4,125E-02 | 0,167 | 0,115 | 0,067 | 0,041 | 0,047 | 0,066 | 0,029 | 0,023 |
| Ruminococcaceae_uncultured | Ruminococcaceae_EU511819 | 4,161E-02 | 0,015 | 0,032 | 0,096 | 0,102 | 0,036 | 0,042 | 1,014 | 1,096 |
| Ruminococcaceae_unclassified | Anaerotruncus_EU006300 | 4,176E-02 | 0,345 | 0,200 | 0,220 | 0,090 | 0,056 | 0,055 | 0,323 | 0,223 |
| Lachnospiraceae_unclassified | Lachnospiraceae_EU511973 | 4,185E-02 | 0,053 | 0,045 | 0,024 | 0,034 | 0,003 | 0,007 | 1,497 | 1,680 |
| Lachnospiraceae_unclassified | Lachnospiraceae_EU505623 | 4,323E-02 | 0,351 | 0,228 | 0,406 | 0,335 | 0,033 | 0,034 | 0,160 | 0,115 |
| Ruminococcaceae_uncultured | Ruminococcaceae_EU006164 | 4,341E-02 | 0,044 | 0,084 | 0,499 | 0,403 | 0,080 | 0,097 | 0,267 | 0,213 |
| Lachnospiraceae_unclassified | Lachnospiraceae_EF406781 | 4,359E-02 | 0,062 | 0,064 | 0,009 | 0,014 | 0,010 | 0,020 | 0,000 | 0,000 |
| Ruminococcaceae_unclassified | Ruminococcaceae_EU507697 | 4,540E-02 | 0,061 | 0,045 | 0,022 | 0,025 | 0,007 | 0,015 | 0,015 | 0,016 |
| Ruminococcaceae_unclassified | Ruminococcaceae_EU457811 | 4,572E-02 | 0,581 | 0,628 | 0,058 | 0,091 | 0,077 | 0,106 | 0,026 | 0,053 |
| Lachnospiraceae_unclassified | Lachnospiraceae_EU504374 | 4,698E-02 | 0,168 | 0,162 | 0,100 | 0,061 | 0,025 | 0,033 | 0,018 | 0,027 |
| Ruminococcaceae_Hydrogenoanaerobacterium | Ruminococcaceae_EU457369 | 4,743E-02 | 0,097 | 0,062 | 0,058 | 0,075 | 0,019 | 0,025 | 0,002 | 0,006 |

SD : standard deviation. Data with different superscript letters are significantly different (p<0.05). The 6 OTUs highlighted in bold have been selected based on the following criteria: their mean abundance is ≥ 1% and their abundance is affected by the POS (fold-change ≥ 7) but not by the INU (fold-change ≤ 6).
