# Supplementary material for: Non Digestible Oligosaccharides Modulate the Gut Microbiota to Control the Development of Leukemia and Associated Cachexia in Mice
Source: PLoS One. 2015 Jun 22;10(6):e0131009. doi: 10.1371/journal.pone.0131009 (PMC4476728; doi:10.1371/journal.pone.0131009)
Supplement: S4 Table — (DOC) [file pone.0131009.s006.doc]

| Nº band | **Closest sequence found in the Genbank database** (% similarity)^1^ | **Accession No.** | **Closest known species** (% similarity)^1^ | **Accession No.** |
| --- | --- | --- | --- | --- |
| 1 | Uncultured bacterium partial 16S rRNA gene, clone 16sms289-1d01 | HE607547.1 | *Bacteroides acidifaciens* gene for 16S ribosomal RNA, partial sequence, strain: SLC8-20 (98%) | AB599950.1 |
| 2 | *Bacteroides dorei* partial 16S rRNA gene, isolate 11-2-D (99%)  *Bacteroides vulgatus* ATCC 8482, complete genome (99%) | HE974919.1  CP000139.1 |  |  |
| 3 | *Prevotella sp.* DJF_B116 16S ribosomal RNA gene, partial sequence (95%) | EU728713.1 | *Prevotella dentalis* DSM 3688 strain DSM 3688 16S ribosomal RNA, complete sequence (94%)  *Prevotella paludivivens* gene for 16S ribosomal RNA, partial sequence, strain: JCM 13650 (93%) | NR_102481  AB547704.1 |
| 4 | *Prevotella sp.* oral taxon 474 clone ID001 16S ribosomal RNA gene, partial sequence (99%) | GU413311.1 | *Prevotella nigrescens* strain ChDC B270 16S ribosomal RNA gene, partial sequence (97%) | AY689230.1 |

**Online Supporting Material**

**Supplemental Table 4** Identification of species found in the DGGE-profiles

^1^Accession numbers were determined by searching in Genbank database using the local BLAST program. We selected as closest relatives those sequences showing maximum identity among those displaying maximum coverage when aligned with sequences from PCR-DGGE amplicons
