# Supplementary material for: Non Digestible Oligosaccharides Modulate the Gut Microbiota to Control the Development of Leukemia and Associated Cachexia in Mice
Source: PLoS One. 2015 Jun 22;10(6):e0131009. doi: 10.1371/journal.pone.0131009 (PMC4476728; doi:10.1371/journal.pone.0131009)
Supplement: S5 Table — (DOCX) [file pone.0131009.s007.docx]

**Online Supporting Material**

**Supplemental Table 5** Fatty acid profile in subcutaneous adipose tissue

|  | **CT** | **BaF** | **BaF-POS** | **BaF-INU** |
| --- | --- | --- | --- | --- |
| **Total fatty acid identified** | **560,69 ± 40,04** | **430,94 ± 44,46** | **486,60 ± 19,87** | **436,96 ± 27,86** |
| **SFA** | **126,77 ± 7,19^a^** | **88,40 ± 7,68^b^** | **100,17 ± 4,86^b^** | **90,68 ± 5,82^b^** |
| C16:0 | 107,59 ± 6,80^a^ | 74,58 ± 8,12^b^ | 84,72 ± 4,34^ab^ | 73,70 ± 5,68^b^ |
| C18:0 | 17,89 ± 1,36^a^ | 12,73 ± 1,19^b^ | 14,25 ± 1,30^ab^ | 15,61 ± 0,74^ab^ |
| C20:0 | 1,07 ± 0,04^ab^ | 0,90 ± 0,06^a^ | 0,98 ± 0,04^ab^ | 1,14 ± 0,05^b^ |
| C22:0 | 0,21 ± 0,01 | 0,19 ± 0,02 | 0,21 ± 0,01 | 0,23 ± 0,01 |
| **MUFA** | **277,85 ± 21,08** | **214,34 ± 19,18** | **238,94 ± 10,30** | **218,36 ± 13,36** |
| C16:1 (*cis*-9) | 38,20 ± 5,52^a^ | 26,05 ± 4,99^ab^ | 27,80 ± 2,27^ab^ | 20,48 ± 2,48^b^ |
| C18:1 (*trans*-9) | 0,46 ± 0,02 | 0,38 ± 0,02 | 0,39 ± 0,02 | 0,39 ± 0,02 |
| C18:1 (*trans*-10) | 0,17 ± 0,01 | 0,14 ± 0,01 | 0,16 ± 0,02 | 0,14 ± 0,01 |
| C18:1 (*trans*-11) | 0,22 ± 0,02^ab^ | 0,19 ± 0,01^a^ | 0,19 ± 0,01^a^ | 0,25 ± 0,02^b^ |
| C18:1 (*cis*-9) | 222,02 ± 14,94^a^ | 173,76 ± 13,05^b^ | 195,26 ± 8,01^ab^ | 183,43 ± 10,44^ab^ |
| C18:1 (*cis*-11) | 16,78 ± 1,56 | 13,82 ± 1,48 | 15,14 ± 0,91 | 13,68 ± 0,90 |
| **PUFA** | **156,08 ± 13,09** | **128,21 ±19,08** | **147,49 ±10,25** | **127,93 ±10,02** |
| C18:2 (*n*-6) | 145,51 ± 11,97 | 120,26 ± 17,85 | 138,48 ± 9,67 | 120,68 ± 9,48 |
| C18:2 (*cis*-9,*trans*-11) | 0,41 ± 0,05 | 0,32 ± 0,02 | 0,35 ± 0,02 | 0,35 ± 0,03 |
| C18:2 (*trans*-10,*cis*-12) | 0,09 ± 0,01^a^ | 0,06 ± 0,004^b^ | 0,08 ± 0,003^a^ | 0,08 ± 0,01^a^ |
| C18:2 (*cis*-9,*cis*-11) | 0,15 ± 0,02 | 0,13 ± 0,02 | 0,15 ± 0,02 | 0,13 ± 0,01 |
| C18:2 (*trans*-11,*trans*-13) | 0,25 ± 0,02 | 0,20 ± 0,02 | 0,24 ± 0,03 | 0,24 ± 0,02 |
| C18:2 (*trans*-9,*trans*-11) | 0,14 ± 0,01 | 0,14 ± 0,01 | 0,14 ± 0,01 | 0,15 ± 0,01 |
| C18:3 (*n*-3) | 7,22 ± 0,79^a^ | 5,08 ± 1,02^ab^ | 5,86 ± 0,62^ab^ | 4,33 ± 0,49^b^ |
| C20:4 (*n*-6) | 1,41 ± 0,15 | 1,22 ± 0,16 | 1,26 ± 0,08 | 1,17 ± 0,04 |
| C20:5 (*n*-3) | 0,12 ± 0,02 | 0,10 ± 0,01 | 0,11 ± 0,02 | 0,10 ± 0,01 |
| C22:5 (*n*-3) | 0,18 ± 0,03 | 0,19 ± 0,02 | 0,21 ± 0,02 | 0,19 ± 0,01 |
| C22:6 (*n*-3) | 0,59 ± 0,08 | 0,51 ± 0,06 | 0,61 ± 0,05 | 0,52 ± 0,02 |
| *n*-6 / *n*-3 ratio | 18,35 ± 0,49^a^ | 22,02 ± 1,61^ab^ | 21,27 ± 1,12^ab^ | 24,35 ± 0,99^b^ |

Fatty acids profile in subcutaneous adipose tissue from mice receiving a saline injection and fed a control diet (CT), mice transplanted with BaF3 cells and fed a control diet (BaF), mice transplanted with BaF3 cells and fed with pectic oligosaccharide (BaF-POS) and mice transplanted with BaF3 cells and fed with inulin (BaF-INU). Values are expressed as mg of fatty acids / g of tissue. Data are mean ± SEM. Data with different superscript letters are significantly different at p<0.05 (One-way ANOVA followed by post hoc Tukey).
