# Supplementary material for: Non Digestible Oligosaccharides Modulate the Gut Microbiota to Control the Development of Leukemia and Associated Cachexia in Mice
Source: PLoS One. 2015 Jun 22;10(6):e0131009. doi: 10.1371/journal.pone.0131009 (PMC4476728; doi:10.1371/journal.pone.0131009)
Supplement: S6 Table — (DOCX) [file pone.0131009.s008.docx]

**Online Supporting Material**

**Supplemental Table 6** Expression of leukemia- and inflammation-related markers in tissues

| **mRNA levels** | **CT** | **BaF** | **BaF-POS** | **BaF-INU** |
| --- | --- | --- | --- | --- |
| **Liver** | | | | |
| Bcr-Abl | n.d. | 1.00 ± 0.05^a^ | 0.87 ± 0.09^ab^ | 0.72 ± 0.20^b^ |
| IL-1β | 1.00 ± 0.09^a^ | 7.42 ± 1.05^b^ | 5.67 ± 0.58^b^ | 6.60 ± 0.83^b^ |
| MCP-1 | 1.00 ± 0.15^a^ | 33.29 ± 2.06^b^ | 32.00 ± 3.31^bc^ | 22.18 ± 3.16^c^ |
| TNF-α | 1.00 ± 0.20^a^ | 12.65 ± 1.54^b^ | 13.27 ± 0.87^b^ | 13.09 ± 1.74^b^ |
| CD68 | 1.00 ± 0.05^a^ | 4.01 ± 0.15^b^ | 3.96 ± 0.21^b^ | 3.87 ± 0.28^b^ |
| **Subcutaneous adipose tissue** | | | | |
| Bcr-Abl | n.d. | 1.16 ± 0.34 | 0.36 ± 0.09 | 1.02 ± 0.26 |
| IL-1β | 1.00 ± 0.97 | 0.28 ± 0.23 | 0.58 ± 0.23 | 0.08 ± 0.05 |
| MCP-1 | 1.00 ± 0.32 | 3.19 ± 0.83 | 2.56 ± 0.37 | 2.95± 0.42 |
| TNF-α | 1.00 ± 0.57 | 2.01 ± 0.29 | 2.13 ± 0.08 | 2.14 ± 0.26 |
| CD68 | 1.00 ± 0.31 | 1.09 ± 0.19 | 1.28 ± 0.18 | 1.17 ± 0.11 |

Mice receiving a saline injection and fed a control diet (CT), mice transplanted with BaF3 cells and fed a control diet (BaF), mice transplanted with BaF3 cells and fed with pectic oligosaccharide (BaF-POS) and mice transplanted with BaF3 cells and fed with inulin (BaF-INU). Data are mean ± SEM. Data with different superscript letters are significantly different at p<0.05 (One-way ANOVA followed by post hoc Tukey). n.d. = not detected.
